# 

**Published:** 1884-01

**Authors:** 


					T(f Mad'ers and "Correspondents.
; Communications intendedifrf ^dfoltatiTTn, and exchange journals and
pkpers should be addressed to the Editor of the American Journal of
Cental Science, 259 N. Eutaw St., Baltimore, Md. All letters relating
t?e received by the editor at least three weeks previous to the first month
on which the number in which it is designed for them to appear, is issued-
j^W The AMeRrCan?J.Q.ViiXA.L,Qfv..D?NXAJL_Sciexce will contain fifty
pages of reading matter in each number, making a volume of about
Six Hwidneck page's,
EXCLUSIM&OT ADYiEKXtSfrMENTS.
X
ERIC MM OURfffi
' IV
&
The Journal is, issued on the first of each monM and*jcontains
r^t less than fifty jfa^Jfof. reading matterin each nurtib(|r, exclu-
s^e- of advertisement^ makiil^% voluiife ;of six, hundred pages
per annum. ? ?? 'fL fc
;/ - >"i 1;
Jn each number will be found Original ^Communications.
Correspondence, Selected ^Arficle&r"Monthly Syanmaiy, Biblio-
graphical Notices and Editorial Matter, and every effort will be
exerted ^o?etide:r the Journal worthy of the patroti&gc of- the .
Cental profession.
A gene?QU s ?? o -ope ration is respectfully solicited frofn the mem-
t>Hfe O^IFprSfeSsiibn^ind ais?the Journal hai now^a printing
office of its own, c&
cation, it will be issuefd at tHe j^uced^^geriptiQiii w"'
$2.30 Fe|c|;^ n?i|i^pjjp*;
domi^nications
taining ? practice
occurring in dental practice.
RATES OF ADVERTISING.
I %'q, ft
idi-tti
:0mm^?faibo[\ $22 50
% " 1 , 71 <P<S> j J I, 00
m. f 5 <>0 g" . .8 60
11 g jg^r' "vr"-- ':&**% i 1 -T ?i?y "vv
is* ? *? ,,
II
* ? ? ,??1"' | fit -<"N' '? .
?30 00 I $50 00
p||H '"
I* M 00
' ???liti90 *'
'jiUjiiL '-^ife^'j'-'-'''*-'-ff
30 00 .
P6#5
' .15 09
rflF^rlgkial communications designed for pu^c^onaM^ks'for^
review, new insirtiments notice/exchanges', 8fe., should be
WPjym gdito-r^ 2^9 N. Eutaw ?t>Baltimore,^d.
?sr-?*?-?-*--?  - ... _
A'Mt ^dvertis&nentsf.^ndr stj bs Crip tionsIsfi6u 1 d be add^seafto
' J . f SNQW0EN& COWMAN, ;i J&J
*,Piit?lMersyf the Ai/i., JviirnaJof Deiital Science,
Bet , C^J-t^&?fe^ERTY
SwAj

				

